# DDX1 is a prognostic biomarker and correlates with immune infiltrations in hepatocellular carcinoma

**DOI:** 10.1186/s12865-022-00533-0

**Published:** 2022-11-30

**Authors:** Mengping Yuan, Jinyong Xu, Shuguang Cao, Shuangshuang Sun

**Affiliations:** 1 Department of Gastroenterology, The Second Affiliated Hospital and Yuying Children’s Hospital of Wenzhou Medical University, Wenzhou, 325000 People’s Republic of China; 2 Department of Pathology, Shenzhen Hyzen Hospital, Shenzhen, 518038 People’s Republic of China; 3 Department of Oncology, The Second Affiliated Hospital and Yuying Children’s Hospital of Wenzhou Medical University, Wenzhou, 325000 People’s Republic of China

**Keywords:** DDX1, Hepatocellular carcinoma, Prognostic biomarker, Tumor microenvironment, Infiltration immune cells, Methylation

## Abstract

Hepatocellular carcinoma (HCC) is one of the leading lethal malignant tumors worldwide. DEAD-box (DDX) family helicases are implicated in numerous human cancers. However, the role of DDX1 in HCC has not yet been fully elucidated. We downloaded gene expression data and clinical information data of HCC from The Cancer Genome Atlas and International Cancer Genome Consortium (ICGC) database and conducted subsequent analyses using the R package and online portal. The results revealed that HCC tissues had higher DDX1 expression compared with either paired or unpaired normal tissues. The increased DDX1 expression was closely related to the advanced pathological grade and histologic grade of HCC. Further analysis suggested that patients with high DDX1 expression contributed to poor prognosis The Cox regression analysis revealed that the expression level of DDX1 was an independent prognostic factor for HCC. In addition, an ICGC cohort was used for external validation. The cBio-Portal, MethSurv, and UALCAN database were used for evaluating the genomic mechanism. Moreover, the Tumor Immune Estimation Resource dataset and QUANTISEQ algorithm revealed that DDX1 expression positively correlates with immune infiltrating cells. We also identified the DDX1-related differentially expressed genes (DEGs) and explored their biological functions by GO, KEGG, and GSEA analyses, which indicated that DDX1 may regulate the progression of HCC. In general, increased DDX1 expression predicts a poor prognosis and drives the progression of HCC.

## Introduction

Hepatocellular carcinoma (HCC) is known to be the fifth most common cancer and is the third cause of cancer-related mortalities worldwide. Thus, it is considered a huge threat to people’s health [1]. Surgical resection and liver transplantation are the most suitable methods for treatment of early-stage HCC. However, for non-resectable patients, radiofrequency ablation (RFA) and stereotactic body radiation therapy (SBRT) are deemed as first-line local treatment options [2, 3]. Nonetheless, many patients with HCC treated with resection or local ablation show early relapse after treatment [4]. In the past decade, immunotherapy has become popular for successfully treating various cancers [5–8]. Interestingly, cumulative evidence revealed that immune-mediated mechanisms are deeply involved in the progression of HCC [9]. This suggests the great potential of immune-based therapies for treating patients with HCC. However, few studies report only satisfactory immunotherapy efficacy for HCC. The failure of immunotherapy could be attributed to the complex composition of the tumor microenvironment (TME). Thus, it is important to understand the tumor immune microenvironment (TIME) for identifying pivotal therapeutic targets and prognostic biomarkers for HCC.

DDX1 is a member of the DEAD-box RNA helicase family and participates in various biological processes, such as mRNA translation, tRNA splicing, rRNA processing, microRNA maturation, and repair of DNA double-strand breaks (DSBs) [10–14]. In addition to its role in transcriptional regulation, a recent study reported that DDX1 interacts with eIF3a and eIF4b to decrease insulin translation [15]. Furthermore, some studies found that DDX1 can inhibit viral replication [16, 17]. Moreover, researchers have found that DDX1 promotes tumorigenesis in various carcinomas, such as retinoblastoma, neuroblastoma, testicular carcinoma, colorectal cancer, and breast cancer [18–21]. Nevertheless, the underlying mechanism of DDX1 in the progression of HCC remains obscure. In this research, we tried to revealed the potential role of DDX1 in the TIME of HCC.

## Materials and methods

### Data collection and analysis

We downloaded the raw RNA sequence data and associated clinical data of patients with HCC from the The Cancer Genome Atlas(TCGA) database. We compared the differential expression of DDX1 between HCC and normal tissues by paired t-test and Mann–Whitney U test. Subsequently, an independent sample t-test was used to analyze the correlation of DDX1 expression with clinicopathologic characteristics, including gender, T stage, pathologic stage, histologic grade, age, alpha fetoprotein (AFP) level and Child-Pugh grade. Analysis and plots were carried out using R packages “rstatix,” “car,” “tidyverse,” “ggplot2,” and “reshape2.” A *P* value of < 0.05 was considered statistically significant.

### Survival analysis and external validation

Based on the average DDX1 expression, the Kaplan Meier (KM) survival curve was used to analyze the prognosis of patients with HCC with different levels of DDX1 mRNA expression. The analyses were conducted using the “survival” and “survminer” packages. Furthermore, we downloaded the RNA-sequencing expression profiles and corresponding clinical information of the liver cancer dataset (RIKEN, JP) from the International Cancer Genome Consortium (ICGC) database. According to the level of DDX1 expression in patient samples, two groups were created. The log-rank test was used to evaluate the difference in overall survival (OS) between the groups. The timeROC analysis was used to compare the predictive accuracy. Log-rank test and Cox regression methods were used to calculate the KM curve P values and hazard ratio (HR) with 95% confidence (CI) intervals.

### Genetic mutation and methylation analysis

The cBioPortal (www.cbioportal.org) is an online exploratory tool for visualizing and analyzing cancer genomic data in multidimensional ways [22]. In this study, we used three different datasets (INSERM, Nat Genet 2015; AMC, Hepatology 2014; TCGA, Firehose Legacy) in cBioPortal to characterize the genomic profiles of DDX1. We categorized the patients into two groups (no mutation and mutation of DDX1) and used the Kaplan–Meier survival curves for evaluating the effect of mutations on survival.

Metasurv (https://biit.cs.ut.ee/methsurv/) provides survival analysis based on CpG methylation patterns [23]. To gain a comprehensive understanding of methylation and prognosis, we accessed the survival rate of the CpG methylation sites in DDX1. Survival was expressed using a Kaplan–Meier (KM) curve. In addition, the UALCAN (ualcan.path.uab.edu/index.html) database was employed to access the correlation of promoter methylation level of DDX1 in HCC with clinicopathological characteristics. Statistical significance was defined as a difference of < 0.05.

### Correlation between DDX1 expression and immune infiltration

With the use of QUANTISEQ algorithm, we determined the association between DDX1 expression and tumor-infiltrating immune cells (TIICs), which including CD4^+^ T cells, CD8^+^ T cells, macrophages and so on. The R package “immunedeconv” was used to reliably estimate immune infiltration [24].

TIMER (https://cistrome.shinyapps.io/timer/) is a public website that analyzes the abundance of TIICs and gene expression across various cancers [25]. According to TIMER dataset, the expression level of DDX1 was associated with the degree of TIICs, including as CD8^+^ T cells, CD4^+^ T cells,macrophages and so on. The above results were executed by the Gene module and visualized by scatter plots. We measured the correlation between TIICs and DDX1 expression using Spearman’s correlation.

### Biological function enrichment analysis

The differentially expressed mRNAs were screened using the Limma package based on the RNA sequence and associated clinical information downloaded from the TCGA database. To correct the false-positive results, the adjusted P-value was analyzed. We set the thresholds for differentially expressed genes (DEGs) as adjusted *P*-value of < 0.05 and |log2 fold change| ≥ 1.5. Kyoto Encyclopedia of Genes and Genomes (KEGG; https://www.kegg.jp/) is a sophisticated database resource for the systematic analysis of gene functions, which links genomic information with higher order functional information [26–28]. Subsequently, the ClusterProfiler R package was utilized to analyze the pathways enriched in DEGs based on Gene Ontology (GO) and KEGG databases.

### Protein interaction screening

STRING (version 11.5) is an online tool that evaluates the interactions of genes [29]. In this study, the STRING dataset was used to search co-expressed genes and construct Protein-Protein Interaction Networks(PPI) networks with an interaction score ≥ 0.15. We identified the top 50 DDX1-interacted proteins. Moreover, using the Similar Genes Detection module of GEPIA (http://gepia.cancer-pku.cn/index.html), we identified the top 100 DDX1-related genes in liver cancer samples from TCGA. We then utilized Online tool (http://bioinformatics.psb.ugent.be/webtools/Venn/) to carry out an intersection analysis between the top 50 DDX1-binding proteins and the top 100 genes related to DDX1 expression. Finally, we investigated the association of DDX1 with the selected genes via the Correlation Analysis module of GEPIA. Results were presented using scatter plots and Pearson correlation coefficient.

### GSEA analysis

RNA-seq data collected from the TCGA database were analyzed using Gene Set Enrichment Analysis (GSEA) to preliminarily classify the genes based on their correlation with DDX1 expression. By GSEA, we carried out a KEGG enrichment analysis to explore the underlying biological roles of DDX1. A false discovery rate (FDR) < 0.05 and a nominal *P*-value < 0.05 were considered statistically significant.

## Results

### Baseline and clinical characteristics of patients with HCC

In all, 374 HCC samples with corresponding clinical features were obtained from the TCGA database. The baseline characteristics and clinical features are summarized in Table 1. The study included 253 males (67.6%) and 121 females (32.4%). Among all participants, 47.5% (n = 177) were ≤ 60 years and 52.5% (n = 196) were > 60 years. As for the histologic grade, 55 (14.9%) patients were grade I, 178 (48.2%) patients were grade II, 124 (33.6%) patients were grade III, and only 12 (3.3%) patients were grade IV. In terms of liver function, there were 219 (90.9%) cases of Child-Pugh Grade A, 21 (8.7%) cases of Grade B, and 1 (0.4%) case of Grade C.

**Table 1:** Clinical characteristics of the patients with LIHC

Characteristic	Levels	Overall (%)
Age, n (%)	≤ 60	177 (47.5)
	> 60	196 (52.5)
Gender, n (%)	Female	121 (32.4)
	Male	253 (67.6)
Race, n (%)	Asian	160 (44.2)
	Black or African American	17 (4.7)
	White	185 (51.1)
Pathologic stage, n (%)	Stage I	173 (49.4)
	Stage II	87 (24.9)
	Stage III	85 (24.3)
	Stage IV	5 (1.4)
T stage, n (%)	T1	183 (49.3)
	T2	95 (25.6)
	T3	80 (21.6)
	T4	13 (3.5)
N stage, n (%)	N0	254 (98.4)
	N1	4 (1.6)
M stage, n (%)	M0	268 (98.5)
	M1	4 (1.5)
Histologic grade, n (%)	G1	55 (14.9)
	G2	178 (48.2)
	G3	124 (33.6)
	G4	12 (3.3)
Adjacent hepatic tissue inflammation, n (%)	None	118 (49.8)
	Mild	101 (42.6)
	Severe	18 (7.6)
AFP(ng/ml), n (%)	≤ 400	215 (76.8)
	> 400	65 (23.2)
Child-Pugh grade, n (%)	A	219 (90.9)
	B	21 (8.7)
	C	1 (0.4)

### Association between DDX1 expression and clinicopathological characteristics in HCC samples

**Fig. 1:**
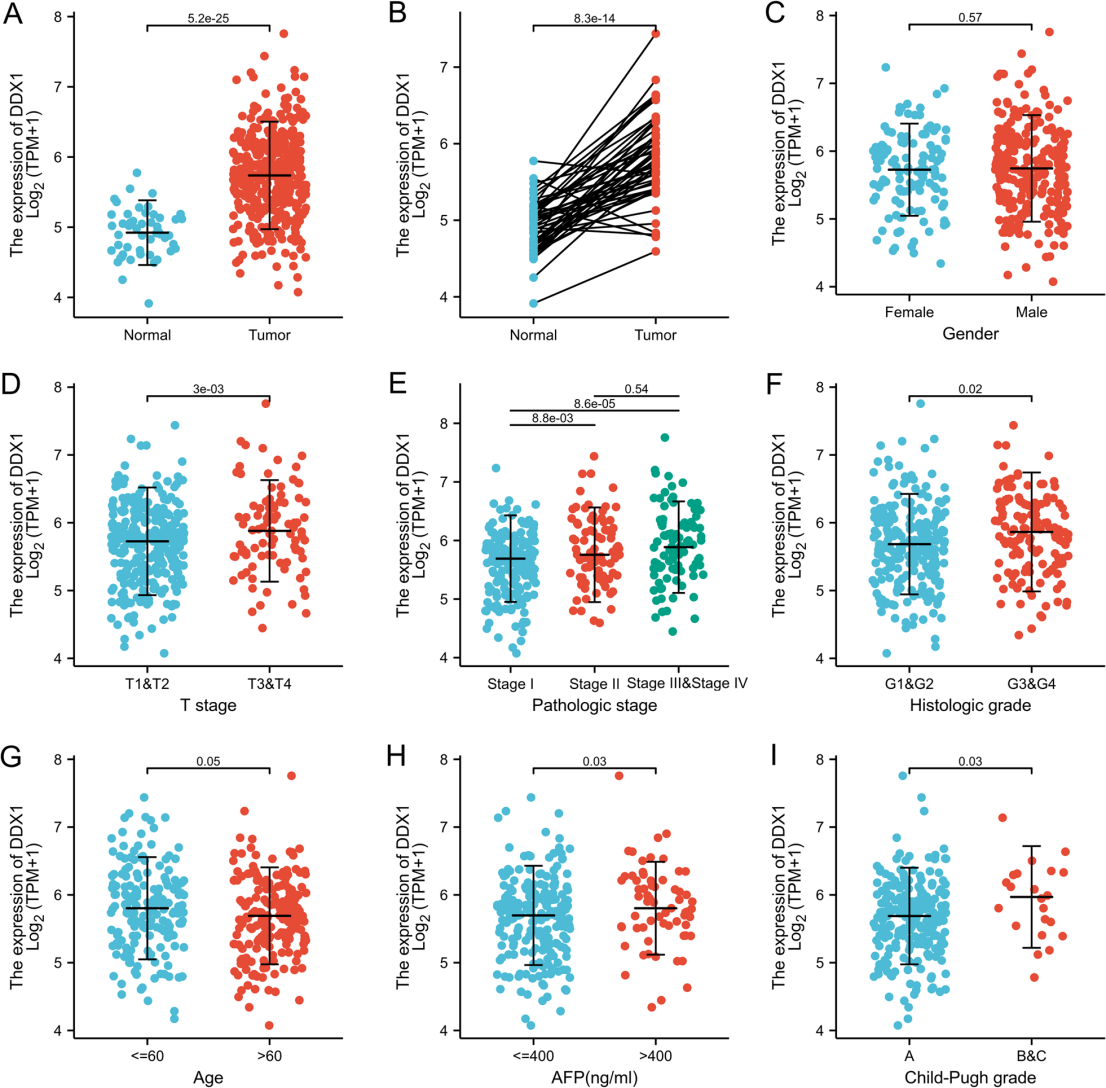
Correlation analysis between DDX1 expression and clinicopathologic features. **A** DDX1 expression in unpaired samples. **B** DDX1 expression in paired sample group; **C** Gender; **D** T stage; **E** Pathologic stage; **F** Histologic grade; **G** Age; **H** AFP level; **I** Child-Pugh grade

Firstly, we analyzed DDX1 expression in patients with HCC. Both in paired and unpaired samples, DDX1 was expressed at higher levels in tumor samples than in normal samples (Fig. 1A, *P* < 0.001 and Fig. 1B, *P* < 0.001). DDX1 expression showed no significant difference between females and males (Fig. 1C, *P* = 0.574). We found that DDX1 expression was positively associated with the T stage of the tumor (Fig. 1D, *P* = 0.003), pathologic stage (Fig. 1E, *P* < 0.001), and histologic grade of HCC (Fig. 1F, *P* = 0.02). Furthermore, the DDX1 expression was closely linked with age, AFP level and Child-Pugh grade of the patients with HCC (Fig. 1G, *P* = 0.047; Fig. 1H, *P* = 0.032; Fig. 1I, *P* = 0.03).

### High DDX1 expression was an independent prognostic factor for HCC

**Fig. 2:**
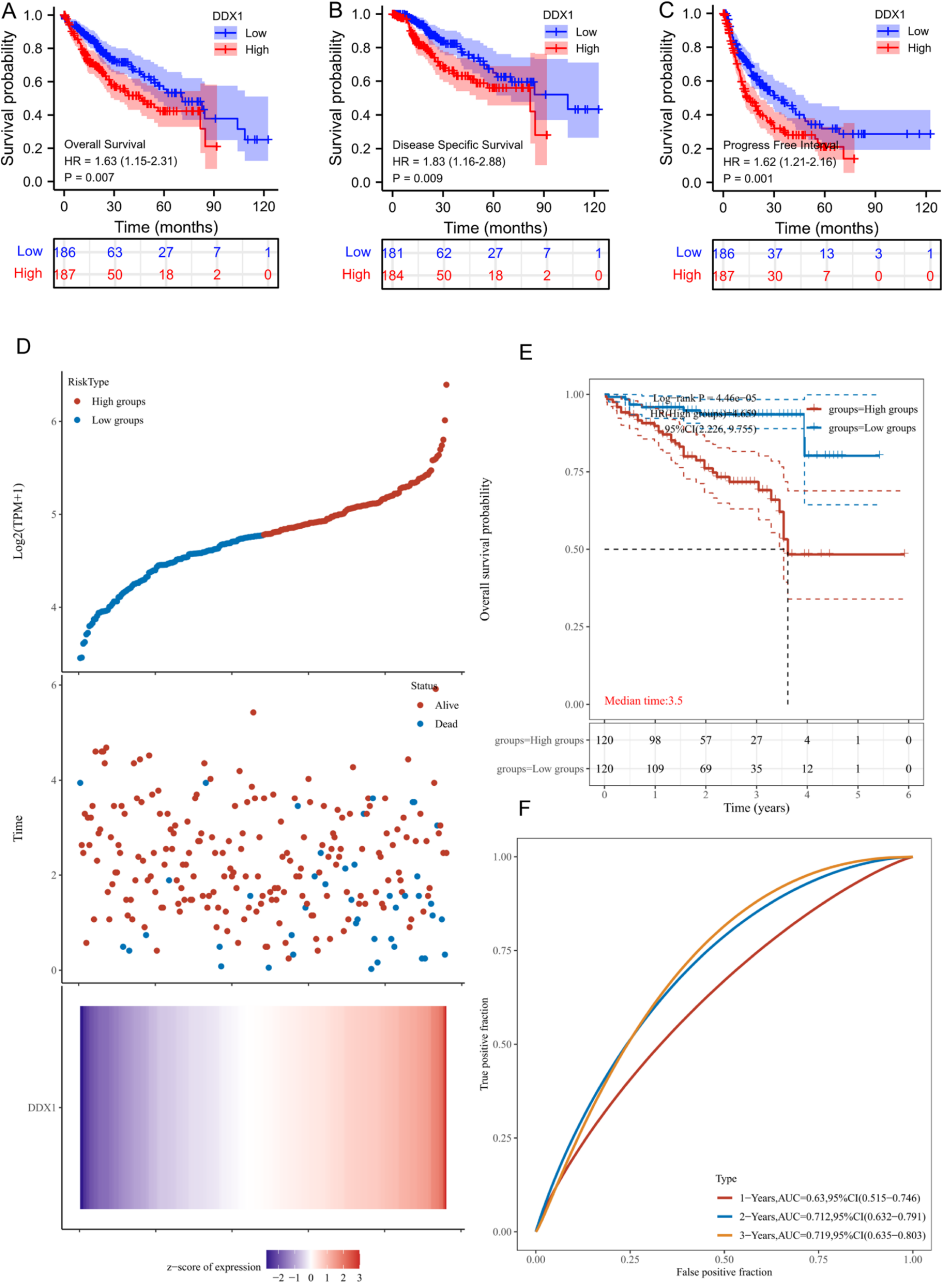
Prognostic analysis and ICGC dataset validation. **A**–**C** Kaplan–Meier survival curve analysis for the effect of DDX1 expression on the prognosis of patients with liver cancer (OS, DSS, and RFI respectively). **D** The DDX1 expression, and survival analyze of the ICGC dataset. **E** Kaplan–Meier survival analysis of the DDX1 signature from ICGC dataset. **F** The ROC curve of the gene

In the TCGA cohort, patients with high-DDX1 expression had significantly shorter OS, disease specific survival(DSS) and progress free interval (PFI) than patients with low-DDX1 expression (Fig. 2A–C). We employed the ICGC database to validate the relation between DDX1 expression and outcome of HCC. According to median DDX1 expression, patients were stratified into high- and low-expression groups. The results revealed that the fatality rate was significantly higher in the high DDX1 expression group compared with the low-expression group (Fig. 2D). In addition, the KM survival analysis indicated that upregulated DDX1 was related to poor patient survival (HR = 4.659, 95% CI = 2.226–9.755, log-rank *P* = 4.46e-05) (Fig. 2E). We further predicted the 1, 2, and 3 year risk of patients with HCC by estimating the area under the curve (AUC) under the ROC curve (1 year, AUC = 0.63; 2 year, AUC = 0.712; 3 year, AUC = 0.719) (Fig. 2F). Overall, the results indicate that DDX1 expression is closely linked to the outcome of patients with HCC.

In addition, univariate Cox regression analysis showed significant relationship of pathologic stage, DDX1 expression (hazard ratio = 1.628, 95% CI = 1.145–2.313, *P* = 0.007), T stage, M stage, and tumor status with poor OS (Table 2). Additionally, multivariate Cox regression analysis identified DDX1 gene expression (HR = 1.822, 95% CI = 1.139–2.915, *P* = 0.012) and tumor status (HR = 1.873, 95% CI = 1.172–2.995, *P* = 0.009) as independent risk factors of total survival for patients with HCC.

**Table 2:** Univariate and multivariate Cox regression analyses of clinical characteristics associated with overall survival

Characteristics	Total (N)	Univariate analysis		Multivariate analysis	
		Hazard ratio (95% CI)	*P* value	Hazard ratio (95% CI)	*P* value
Age	373				
≤ 60	177	Reference			
> 60	196	1.205 (0.850–1.708)	0.295		
Gender	373				
Female	121	Reference			
Male	252	0.793 (0.557–1.130)	0.200		
Pathologic stage	349				
Stage I & Stage II	259	Reference			
Stage III & Stage IV	90	2.504 (1.727–3.631)	**< 0.001**	1.341 (0.182–9.864)	0.773
Histologic grade	368				
G1&G2	233	Reference			
G3&G4	135	1.091 (0.761–1.564)	0.636		
DDX1	373				
Low	186	Reference			
High	187	1.628 (1.145–2.313)	**0.007**	1.822 (1.139–2.915)	**0.012**
AFP(ng/ml)	279				
≤ 400	215	Reference			
> 400	64	1.075 (0.658–1.759)	0.772		
T stage	370				
T1&T2	277	Reference			
T3&T4	93	2.598 (1.826–3.697)	**< 0.001**	1.833 (0.248–13.569)	0.553
N stage	258				
N0	254	Reference			
N1	4	2.029 (0.497–8.281)	0.324		
M stage	272				
M0	268	Reference			
M1	4	4.077 (1.281–12.973)	**0.017**	1.856 (0.427–8.075)	0.410
Child-Pugh grade	240				
A	218	Reference			
B&C	22	1.643 (0.811–3.330)	0.168		
Residual tumor	344				
R0	326	Reference			
R1&R2	18	1.604 (0.812–3.169)	0.174		
Tumor status	354				
Tumor free	202	Reference			
With tumor	152	2.317 (1.590–3.376)	**< 0.001**	1.873 (1.172–2.995)	**0.009**

Bold values indicates* p* < 0.05

### DDX1 genetic mutation and methylation in patients with HCC

**Fig. 3:**
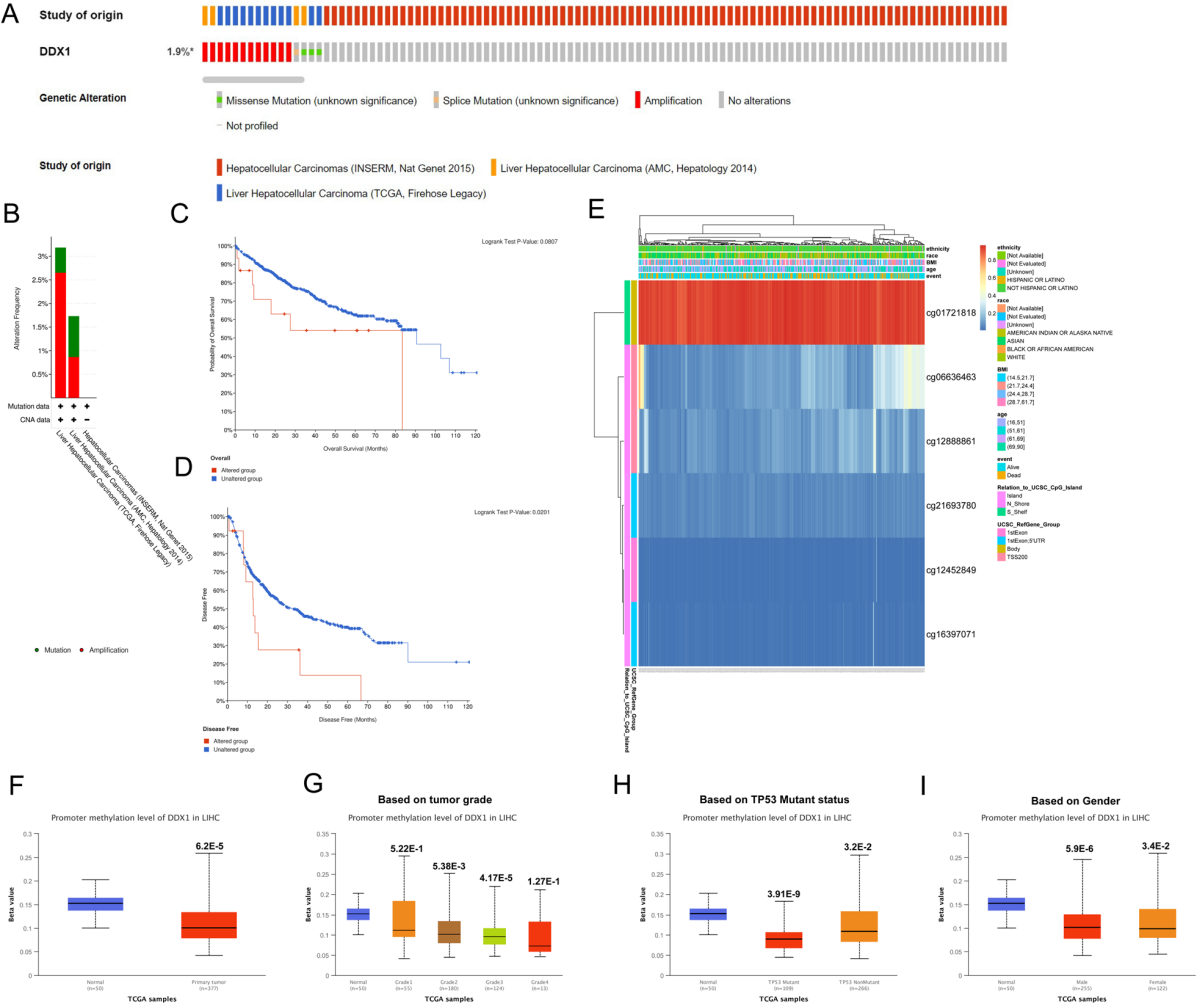
Genetic alteration and methylation of DDX1 in HCC. **A** OncoPrint visual summary of alteration in DDX1. **B** Summary of alterations in DDX1 in HCC based on three datasets. **C**, **D** Comparison of the survival prognosis between DDX1 mutation and unmutation groups. **E** Visualization of six CpG sites on DDX1 with methylation level. **F**–**I** Relation between DDX1 promoter methylation in HCC and normal tissues and clinicopathological characteristics: **F** normal versus primary tumor, **G** tumor grade, **H** TP53 mutation status, and **I** gender

In all, we included data of 916 patients across three datasets (Fig. 3A) and found that the DDX1 gene alteration rate ranged from 1.73 to 1.81% (Fig. 3B). The mutated and not mutated groups showed no difference in OS (log-rank *P* = 0.0807) (Fig. 3C). However, the DFS rates of the two groups differed significantly (Fig. 3D log-rank *P* = 0.0201). Next, we detected the DNA methylation levels of DDX1, with the prognostic value of each single CpG by using MethSurv. The headmap graph shown in Fig. 3E showed the methylation of the six CpG sites in LIHC. Of these, cg01721818 methylation was the highest. In addition, the higher the DNA methylation level of cg01721818 and cg21693780, the worse the prognosis (Table 3). Moreover, subgroup analysis of the UALCAN database showed that the promoter methylation of DDX1 was significantly lower in patients with LIHC than in normal controls in subgroups based on grade, gender, and TP53 mutant status (Fig. 3F–I).

**Table 3:** Effect of hypermethylation level on prognosis in LIHC

CpG	HR	CI	*P*-value
cg01721818	1.948	(1.209;3.14)	0.006177134
cg06636463	1.079	(0.765;1.522)	0.664338648
cg12452849	1.19	(0.842;1.681)	0.325095456
cg12888861	1.544	(0.984;2.423)	0.058794328
cg16397071	0.87	(0.617;1.226)	0.425388277
cg21693780	1.519	(1.071;2.155)	0.019020876

### DDX1 is correlated with tumor immune infiltration in HCC

**Fig. 4:**
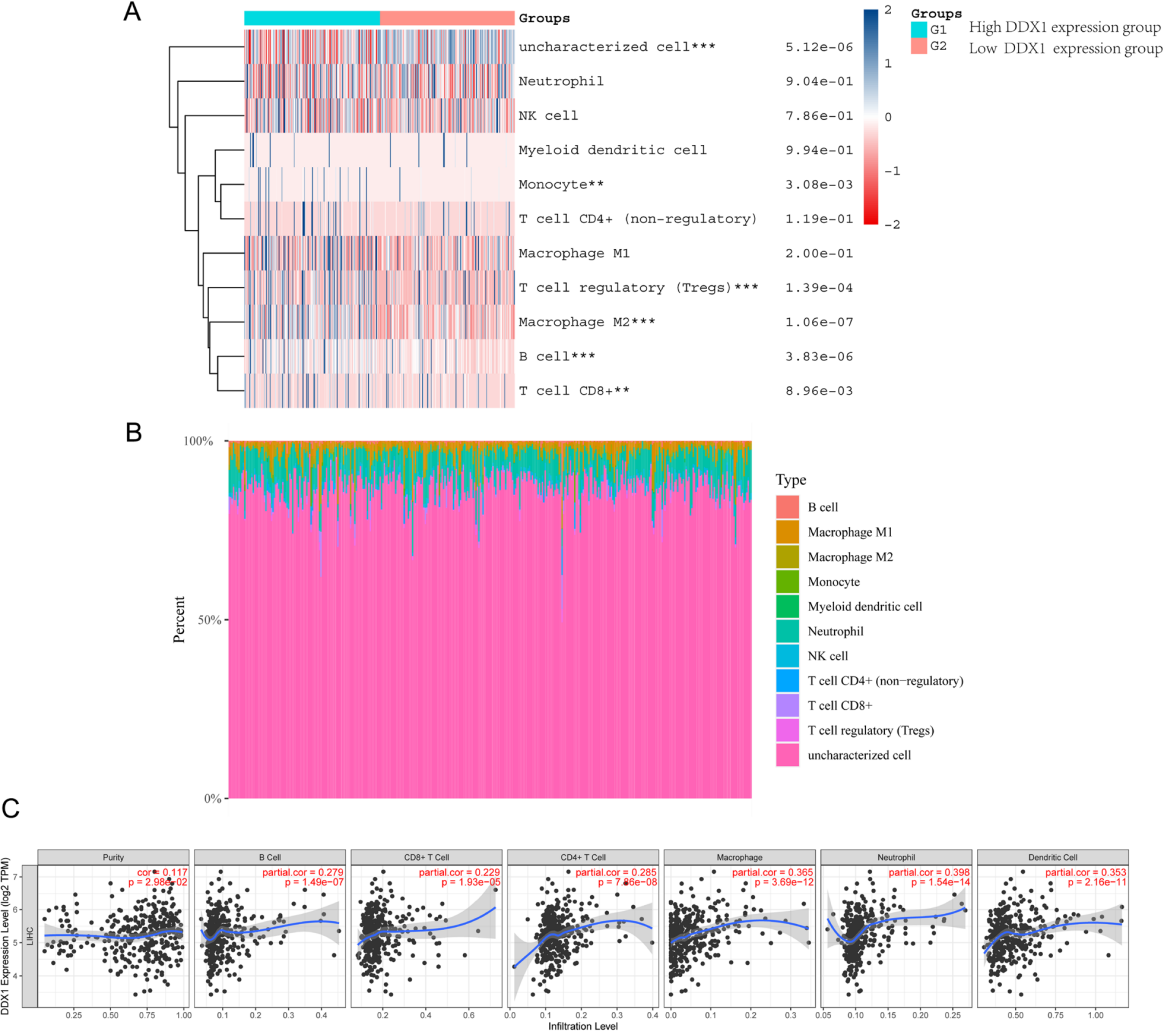
**A**, **B** The different proportions of 10 subtypes of immune cells in tumor sumple expressing high and low level of DDX1. **C** Association of DDX1 expression with immune infiltration levels

We assessed the expression of 10 immune cell subtypes in the high and low DDX1 expression groups using the QUANTISEQ algorithm (Fig. 4A, B). We found that DDX1 expression was related to CD8^+^ T cell, B cell, M2 macrophages, Tregs, and monocytes. The immune infiltration scores of M2 macrophages and Tregs were higher in the high DDX1 expression group compared with the low DDX1 expression group. Moreover, we analyzed the relationshipof DDX1 expression the immune infiltration cells via TIMER. As depicted in Fig. 4C, the expression level of DDX1 was positively related with B cells (r = 0.279, *P*-value = 1.49e-07), CD8^+^ T cells (r = 0.229, *P*-value = 1.93e-05), CD4^+^ T cells (r = 0.285, *P*-value = 7.86 × 10^− 8^), macrophages (r = 0.365, *P*-value = 3.69 × 10^− 12^), neutrophils (r = 0.398, *P*-value = 1.54 × 10^− 14^), and dendritic cells (r = 0.353, *P*-value = 2.16 × 10^− 11^). These results indicate that DDX1 plays a pivotal role in immune infiltration in HCC.

### Screening of DEGs and functional enrichment analysis

**Fig. 5:**
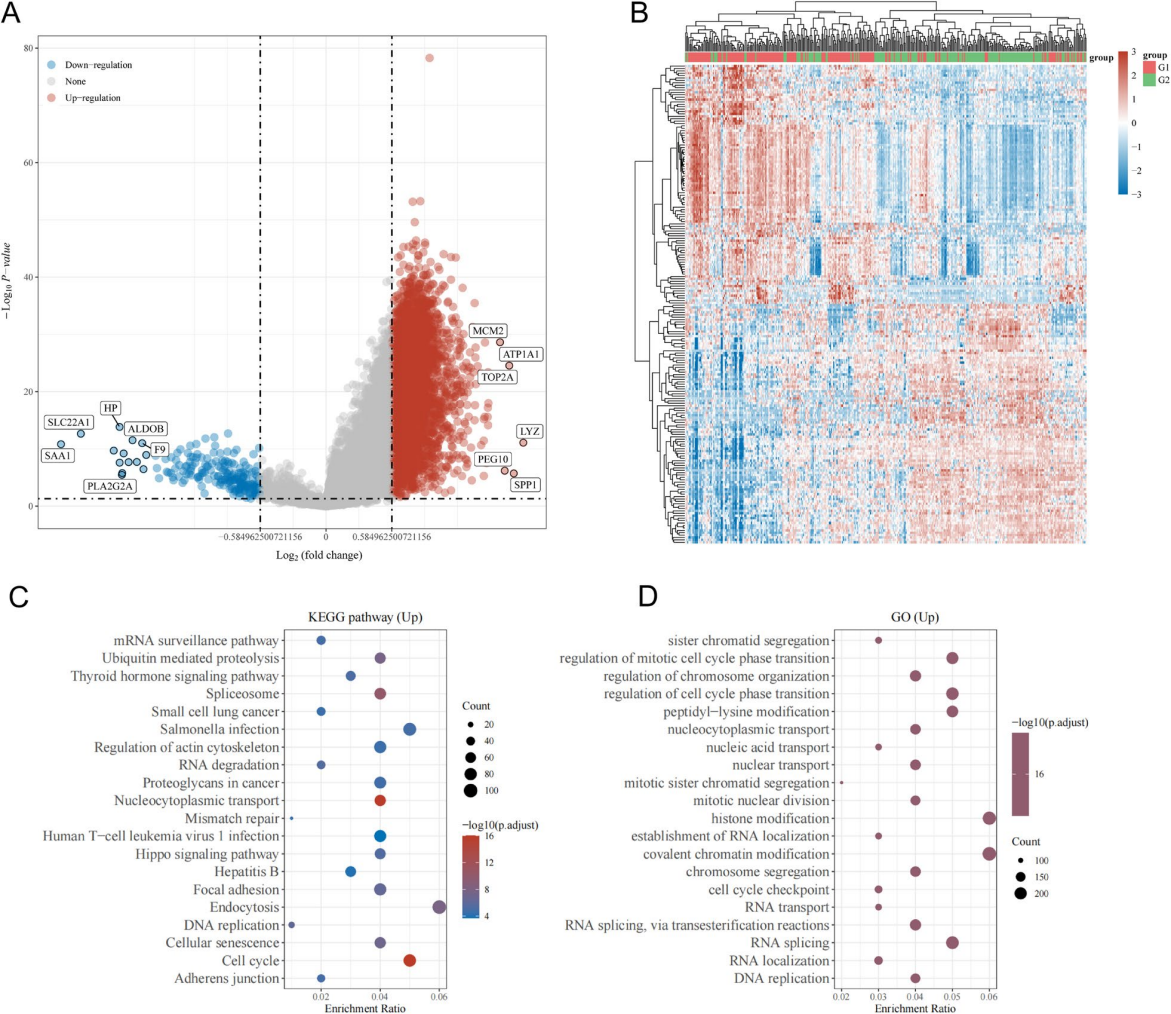
Enrichment analysis of DDX1 expression-correlated DEGs in HCC. **A** Volcano plot of DEGs between high and low DDX1 expression groups. **B** Clustering analysis heatmap of DDX1 expression-correlated DEGs. **C**, **D** KEGG and GO analyses of upregulated DEGs

To further investigate the biological functions of DDX1 in HCC, we identified the DEGs. Figure 5A shows the volcano plot for DEGs, of which 4212 were upregulated and 205 were downregulated. Using a hierarchical clustering analysis of DEGs, heatmaps were created to observe the genes in similar samples (Fig. 5B). To explore the potential biological roleof upregulated DEGs in HCC, we used GO and KEGG enrichment analyses. KEGG enrichment results demonstrated that the upregulated DEGs were mainly enriched in the spliceosome, proteoglycans in cancer, focal adhesion, cell cycle, cellular senescence, and endocytosis (Fig. 5C). Next, GO enrichment analysis of upregulated DEGs indicated that they were correlated with cell cycle regulation, RNA editing including RNA splicing, covalent chromatin modification, histone modification, regulation of cell cycle phase transition, and regulation of mitotic cell cycle phase transition (Fig. 5D).

### PPI network construction and correlation analysis

**Fig. 6:**
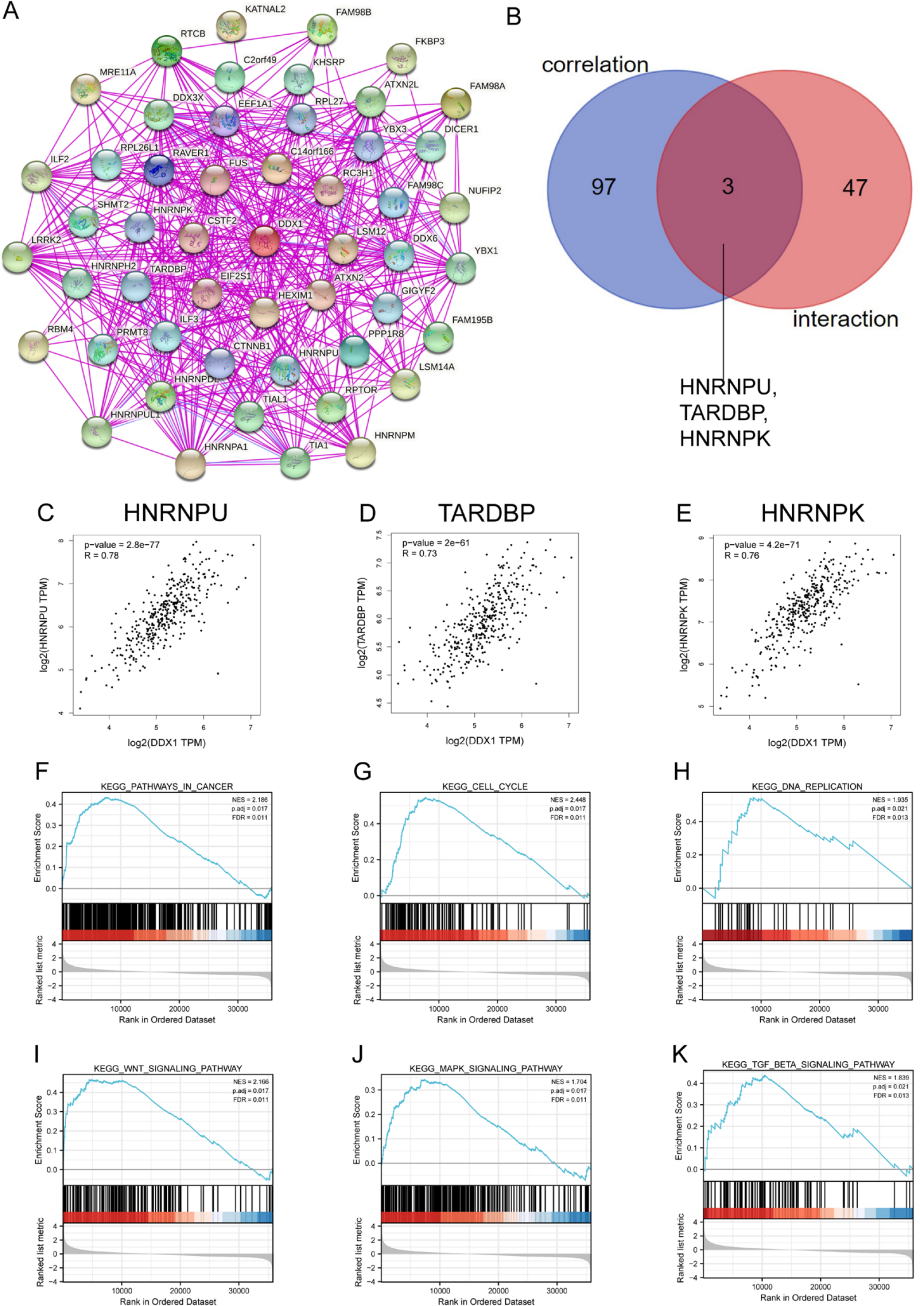
DDX1-related gene enrichment analysis and GSEA analysis. **A** STRING website was used to extract the top 50 DDX1-binding proteins which were supported by available experiments. **B** Intersection analysis of DDX1-binding and correlated genes. **C**–**E** Using the GEPIA approach, we obtained the top 100 DDX1-correlated genes in TCGA and analyzed the expression correlation between DDX1 and selected targets, including HNRNPU, TARDBP, HNRNPK **F** Pathways in cancer, **G** cell cycle, **H** DNA replication, **I** Wnt signaling pathway, **J** MAPK signaling pathway, and **K** TGF-β signaling pathway

To determine the intrinsic role of DDX1 in HCC tumorigenesis, we used STRING to identify the top 50 DDX1-binding proteins (Fig. 6A). Subsequently, GEPIA was used to identify the top 100 genes correlating with DDX1 expression. We conducted the intersection analysis of the above two groups and found HNRNPU (heterogeneous nuclear ribonucleoprotein U), TARDBP (TAR DNA-binding protein), and HNRNPK (heterogeneous nuclear ribonucleoprotein K) as common factors (Fig. 6B). The DDX1 expression was positively correlated with HNRNPU (Fig. 6C, R = 0.78, *P*-value = 2.8e-77), TARDBP (Fig. 6D, R = 0.73, *P*-value = 2e-61), and HNRNPK (Fig. 6E, R = 0.76, *P*-value = 4.2e-71).

### Identification of DDX1-related signaling pathways by GSEA analysis

We performed a GSEA analysis to identify the DDX1-related signaling pathways involved in HCC. The results showed that DDX1-associated DEGs were significantly enriched in cancer pathway (Fig. 6F–K), such as Pathways in cancer [normalized enrichment score (NES) = 2.186, adjusted *P*-value = 0.017, FDR = 0.011], MAPK signaling pathway (NES = 1.704, adjusted *P*-value = 0.017, FDR = 0.011), Wnt signaling pathway (NES = 2.166, adjusted *P*-value = 0.017, FDR = 0.011), TGF-β signaling pathway (NES = 1.839, adjusted *P*-value = 0.021, FDR = 0.013), and DNA replication (NES = 1.935, adjusted *P*-value = 0.021, FDR = 0.013). Interestingly, DDX1-associated DEGs were associated with cell cycle (NES = 2.448, adjusted *P*-value = 0.017, FDR = 0.011). These results suggest that DDX1-associated DEGs may participate in these signaling pathways to modulate tumor progression.

## Discussion

The DEAD-box RNA helicase family is known to play critical roles in various RNA metabolic processes. Recent studies indicate that DDX members are dysregulated in multiple cancers and function as key players in tumor progression [30]. Hu et al. identified that DDX51 regulates cellular proliferation in esophageal squamous cell carcinoma through the PI3K/AKT/mTOR pathway [31]. Jiang et al. proved that knock-down of DDX46 caused a significant reduction in cell invasion and migration in osteosarcoma [32]. DDX1 activates the transcription of 12p stem cell genes in testicular tumorigenesis [19]. Tanaka et al. proved the role of DDX1 in promoting colorectal tumorigenesis in vitro and in vivo [20]. Researchers have proved that high DDX1 expression is associated with improvement in local control, distant metastatic-free survival, and OS when compared with low DDX1 expression in node-negative and early-stage patients with breast cancer [33]. However, the role of DDX1 in HCC is not yet determined. Therefore, in this study, we focused on determining the potential prognostic value of DDX1 in HCC.

We acquired HCC samples from the TCGA database to explore the role of DDX1 in HCC progression. The results reveal that high DDX1 expression is associated with a poor prognosis of HCC. On analyzing the relationship between DDX1 and clinicopathologic features we found that DDX1 may serve as an adverse prognostic factor in HCC. Patients with high DDX1 expression may present an advanced T stage, histologic grade, and pathological stage.

According to molecular profiles and clinical outcomes, HCC has a highly heterogeneous nature, which present a formidable challenge to an accurate diagnosis and treatment [34]. Luckily, previous studies have proved that molecular subtype stratification could overcome the hurdles caused by tumor heterogeneity [35, 36]. In this research, 240 HCC samples downloaded from ICGC dataset were divided into high and low DDX1-expression subgroups, the KM survival analysis revealed that patients with high levels of DDX1 are at higher risk of suffering a poor prognosis. This results were consistent with the findings from TCGA dataset.

As liver carcinoma is typically associated with high malignancy, it is usually diagnosed at a late stage, posing a challenge for radical surgery. In order to improve the outcome of HCC, early diagnosis and treatment are crucial. As we known, tumorigenesis has been demonstrated to result from multiple gene mutations, patients with gene mutations are known to have a poor prognosis, In this research,we accessed the alteration percentage of DDX1 in HCC. After analyzing three independent datasets, we found the percentage of DDX1 alteration to be 1.9%. Furthermore, no significant differences was observed in OS between the mutation and unmutation DDX1 groups. In addition to gene mutations, numerous epigenetic changes, such as DNA methylation and histone modifications, contribute to tumor development [37–39]. Nowadays, since DNA methylation is tissue-specific and stable, detecting abnormal DNA methylation in liquid biopsies has been shown to be a promising biomarker for cancer diagnosis [40, 41]. A previous study indicated that HCC is caused by DNA methylation [42]. For example, Kuo et al. found that patients with higher IRAK3 methylation had worse prognosis [43]. However, no study has examined the relationship between DDX1 methylation and the oncologic outcome of HCC. Hence, in this study, we carried out methylation analysis and evaluated the correlation of methylation level with prognosis. We identified six CpG sites and two of them with hypermethylation were associated with poor prognosis. Besides, a previous study demonstrated that the hypomethylation status of oncogenes could also promote tumor development [44]. In this study, using UALCAN we found that HCC tissues had lower levels of DDX1 promoter methylation than normal tissues (*P* < 0.05). Further analysis indicated that the high tumor grade was linked to low promoter methylation levels. Thus, DDX1 methylation examination has the potential to be developed as a screening tool for predicting tumor status and progression; however, further in vivo and in vitro experiments are needed.

TME is a complicated assembly of the tumor, immune, stromal, and extracellular components [45]. Previous studies revealed that TME facilitates the progression of HCC, thus indicating that it could be exploited as a potential therapeutic target [46]. The importance of immune cell infiltration in the TME has been recognized for various solid tumors [47, 48]. In this study, we determined the association between DDX1 expression and immune cell infiltration in HCC through the TIMER database. The results revealed that DDX1 is positively associated with dendritic cells, B cells, macrophages, and T cells. In addition, we verified the positive correlation between DDX1 expression and immune cell infiltration using the QUANTISEQ algorithm. M2 macrophages and Tregs were higher in the high DDX1 expression group compared with the low DDX1 expression group. Several studies indicate that macrophages are essential components of the TME and play key roles in tumor progression [49]. Liu et al. [50] found that M2 macrophages target miR-149-5p/MMP9 signaling pathway thereby facilitating HCC progression. Yeung et al. demonstrated that M2 macrophages are related to the adverse prognosis of liver cancer and promote HCC invasion by promoting Epithelial mesenchymal transformation(EMT) [51]. Another research revealed that M2-polarized macrophages promote EMT of HCC cells and accelerate tumor progression through the TLR4/STAT3 signaling pathway [52]. Similarly, Tregs are also critical components of TME. Jiang et al. proved that Tregs are closely associated with the prognosis of HCC. They secrete TGF-β1 which triggers EMT, thereby enhancing tumor invasiveness [53]. Shen et al. [54] put forth that TGF-β could drive Treg cell polarization to promote the progression of HCC. These results suggest that DDX1 plays a vital role in the TIME of HCC. However, additional experimental and theoretical studies are needed to validate the relationship between DDX1 and tumor infiltration.

To explore the potential biological functions of DDX1, we identified the DEGs in HCC samples and conducted GO and KEGG enrichment analyses. The results showed that DDX1 was mainly related to cell cycle and RNA editing. In addition, since DEAD box proteins function by interacting with other proteins, they are susceptible to being regulated by their partners and their microenvironment [55]. Hence, using STRING and GEPIA database, we extracted 50 DDX1-binding proteins and the top 100 DDX1-related genes. Intersection analysis of the two groups identified HNRNPU, TARDBP, and HNRNPK as the common hits. TARDBP is an RNA-binding protein involved in the cell cycle of HCC tumor cells, and its expression level is related to an advanced stage and high grade of HCC [56]. The CDK2 protein is known as an essential role in cell cycle regulation [57]. Liang et al. have found HNRNPU enhances CDK2 transcription, thereby promoting HCC development [58]. GSEA analysis also identified significant KEGG pathways associated with the cell cycle.

## Conclusion

In summary, this study is the first to identify DDX1 as a diagnostic and prognostic biomarker of HCC. The results indicate that DDX1 has an important role in TIME and is involved in the regulation of the cell cycle of HCC. Therefore, targeted DDX1 therapy is a potential treatment strategy for patients with HCC.

## Data Availability

All data analyzed in the present study was obtained from TCGA and ICGC.
